# Loss of sympathetic innervation to islets of Langerhans in canine diabetes and pancreatitis is not associated with insulitis

**DOI:** 10.1038/s41598-020-76091-5

**Published:** 2020-11-05

**Authors:** Chen Gilor, Jully Pires, Rachel Greathouse, Rebecca Horn, Mark O. Huising, Stanley L. Marks, Brian Murphy, Amir Kol

**Affiliations:** 1 Department of Veterinary Medicine and Epidemiology, College of Veterinary Medicine, University of California, One Shields Ave., Davis, CA 95616 USA; 2 Department of Small Animal Clinical Sciences, College of Veterinary Medicine, University of Florida, 2015 SW 16th Ave, Gainesville, FL 32610 USA; 3 Department of Pathology, Microbiology and Immunology, College of Veterinary Medicine, University of California, One Shields Ave., Davis, CA 95616 USA; 4 Department of Neurobiology, Physiology and Behavior, College of Biological Sciences, University of California, One Shields Ave., Davis, CA 95616 USA

**Keywords:** Type 1 diabetes, Diabetes complications

## Abstract

Canine diabetes mellitus (DM) affects 0.6% of the canine population and yet, its etiology is poorly understood. Most affected dogs are diagnosed as adults and are insulin-dependent. We compared pan-leukocyte and sympathetic innervation markers in pancreatic islets of adult dogs with spontaneous DM (sDM), spontaneous pancreatitis (sPanc), both (sDMPanc), toxin-induced DM (iDM) and controls. We found evidence of decreased islet sympathetic innervation but no significant infiltration of islets with leukocytes in all disease groups. We show that loss of sympathetic innervation is ongoing in canine DM and does not necessarily precede it. We further found selective loss of islet-associated beta cells in dogs with sDM and sDMPanc, suggesting that collateral damage from inflammation in the exocrine pancreas is not a likely cause of DM in these dogs. The cause of this selective loss of beta cells needs to be further elucidated but overall, our findings are not supportive of an autoimmune process as a cause of sDM in adult dogs. The loss of sympathetic innervation in sPanc in dogs that do not suffer from DM links the disease in the exocrine pancreas to a pathological process in the endocrine pancreas, suggesting pancreatitis might be a potential precursor to DM.

## Introduction

Diabetes mellitus (DM) is a common disease in dogs affecting about 0.6% of dogs in the USA with increasing prevalence^1^. Like Type 1 diabetes mellitus (T1DM) human patients, most diabetic dogs are insulin-dependent and ketosis-prone, and insulin resistance does not play a significant role in the disease process^2^. In both species, DM can be associated with inflammation in the exocrine pancreas (pancreatitis) although the cause and effect relationship is uncertain^3–7^. As in human T1DM, canine DM is associated with significant selective loss of beta cells in pancreatic islets^8^. Because of these similarities, canine DM has often been labeled as T1DM, although evidence for the hallmark of T1DM, autoimmune beta cell destruction, has been identified in only a small fraction of canine cases^2,7,9^. Therefore, we set out to further characterize the pathogenesis of canine DM and examine similarities, or lack thereof, to T1DM in humans. Specifically, we aimed to (1) assess the frequency of insulitis (inflammation within the islets of Langerhans) based on current criteria^10^ (2) assess changes in sympathetic innervation of islets^11^ (3) compare these findings between dogs diagnosed with spontaneous DM (sDM) lacking histological evidence of pancreatitis, dogs diagnosed concurrently with spontaneous DM and pancreatitis (sDMPanc), dogs with pancreatitis and no DM, and purpose-bred research dogs with alloxan-streptozotocin-induced DM (iDM).

In humans with T1DM and in rodent models, pancreatic pathology is characterized not only by a predominately lymphocytic insulitis, but also by early, sustained, islet-selective loss of sympathetic innervation^11^. The pathogenesis of this neuropathy is unclear but it appears to be a result of the combination of beta cell loss together with the inflammatory milieu of the autoimmune process within the islet. This neuropathy is not observed in Type 2 DM or when beta cells are destroyed by a toxin in rodent models^11–13^. It has not been determined whether the loss of sympathetic innervation in T1DM occurs secondary to other inflammatory diseases in which lymphocytes do not necessarily predominate (such as pancreatitis). Loss of sympathetic innervation to islets might serve as a marker for prior autoimmune destruction of insulin-producing beta cells.

In humans with T1DM, the number of affected islets and the number of infiltrating immune cells in each affected islet varies but is usually low, especially in patients diagnosed in middle age^10^. There are no established criteria for diagnosis of insulitis in canine DM, and until recently, none existed for T1DM. In a recent consensus guideline, the threshold for diagnosis of insulitis in people with T1DM has been established as a minimum of three affected islets and a minimum of 15 CD45+ (leukocyte common antigen) cells per islet (either intra-islet, or at the periphery of the islet)^10^. Importantly, these standards were established based on a comparison to a non-diabetic control group because it is recognized that background inflammation (as well as presence of resident lymphocytes) in the exocrine pancreas is common in humans. To our knowledge, these standards have not been applied to histopathological samples from canine DM to date. To that end, we aimed to compare the number and distribution of CD45+ leukocytes in pancreata from dogs with sDM and sDMPanc to dogs that have no evidence of either DM or pancreatitis and dogs with pancreatitis but no DM.

In dogs and human patients, DM is often diagnosed concurrently with pancreatitis . The association between DM and pancreatitis in dogs and in people is complex and establishing cause and effect is difficult^4,9^. There is evidence that at least in a subset of affected dogs, pancreatitis precedes and increases the risk of DM. Some histopathological evidence of chronic pancreatitis is found in most dogs that die from diseases unrelated to the pancreas and that do not present with clinical signs associated with pancreatitis or DM. In one study, 90% of dogs that died of a variety of causes had histopathological evidence of pancreatitis (acute and chronic)^14^. Even if pancreatitis does not cause DM directly, its high prevalence confounds the interpretation of histopathological findings of inflammation in the endocrine pancreas and should be considered at least as “background” when assessing the degree or presence of insulitis.

We hypothesized that in diabetic dogs, leukocytes will infiltrate islets of Langerhans at a higher frequency compared to non-diabetic dogs. We also hypothesized that sympathetic innervation of islets will be decreased in diabetic dogs, but not when DM is associated with pancreatitis or in cases of toxin-induced DM. Finally, we hypothesized that in pancreatitis, DM is the result of indiscriminate collateral damage to islets. Therefore, in dogs with DM and concurrent pancreatitis, beta cell loss would be associated with loss of other endocrine cell types, leading to a greater reduction in endocrine cell mass in these dogs compared to dogs suffering from DM alone.

Here we report for the first time on sympathetic denervation of pancreatic islets in adult dogs, not only with DM, but also in dogs with pancreatitis and no DM. We also show that sympathetic denervation is ongoing in canine DM and as previously reported, we found no evidence for insulitis in canine DM. Together, these findings are not supportive of an autoimmune process as a cause of DM in adult dogs. We further found selective loss of islet-associated beta cells in dogs with spontaneous DM, with or without pancreatitis, suggesting that collateral damage from inflammation in the exocrine pancreas is not the cause of DM in these dogs. The cause of this selective loss of beta cells needs to be further elucidated.

## Results

Age, sex and body weight are presented in Table 1. Body condition scores were unavailable for most dogs and therefore not reported. Except for the iDM group which included only purpose-bred Beagles, there was no dominant breed in any group, with a total of 18 breeds represented and 14 mix-breed dogs in the four groups (Table S3).

**Table 1:** Study population demographics.

Group	Age (years)	Sex	Body weight (kg)
Controls (N = 5)	4.6 ± 2.6^a^	FS = 1, FI = 0	27.4 ± 18.8
	4 (0.8–7.0)	MC = 4, MI = 0	23.0 (2.4–51.7)
sDM (N = 11)	10.2 ± 2.9^b^	FS = 4, FI = 1	26.0 ± 15.8
	10 (5–14)	MC = 5, MI = 1	28.2 (4.4–52.0)
sPanc (N = 10)	7.9 ± 3.8	FS = 6, FI = 0	20.2 ± 8.1
	7.0 (0.8–14)	MC = 4, MI = 0	20.2 (10.4–30.0)
sDMPanc (N = 10)	9.7 ± 3.0	FS = 6, FI = 1	21.5 ± 18.0
	10.0 (4.0–14)	MC = 2, MI = 1	12.9 (5.0–60.0)
iDM (N = 7)	3.0 ± 0	FS = 0, FI = 0	9.9 ± 1.5
		MC = 7, MI = 0	10.5 (7.5–11.2)

Data are presented as Mean ± SD; Median (range). Different superscripts indicate significantly different values (p ≤ 0.05).

*DM* diabetes mellitus, *sDM* spontaneous DM, *sDMPanc* spontaneous DM and concurrent pancreatitis, *sPanc* spontaneous pancreatitis, *iDM* toxin-induced DM, *FS* female spayed, *FI* female intact, *MC* male castrated, *MI* male intact.

**Figure 1:**
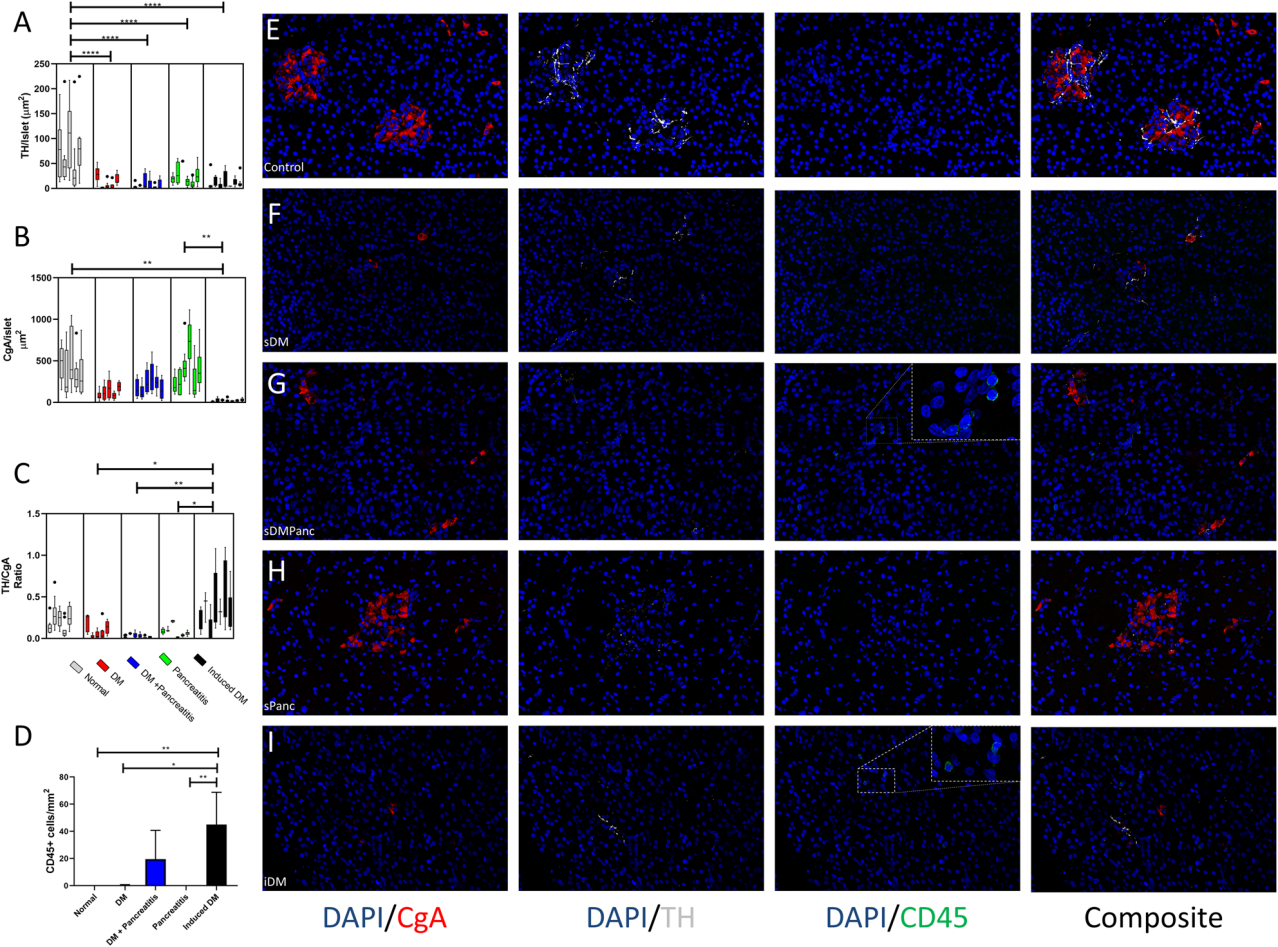
Loss of sympathetic innervation in dogs with pancreatic disease. *DM* diabetes mellitus, *sDM* spontaneous DM, *sDMPanc* spontaneous DM and concurrent pancreatitis, *sPanc* spontaneous pancreatitis, *iDM* toxin-induced DM. (**A**) Sympathetic innervation (indicated by TH area per islet), is markedly reduced in all disease groups compared with control dogs. (**B**) Islet size (indicated by CgA area per islet), is markedly decreased in iDM dogs vs. controls and sPanc groups. (**C**) When TH loss is controled for endocrine cell loss, iDM dogs have increased TH-to-CgA ratio compared with all other disease groups. (**D**) In iDM dogs, more leukocytes (CD45+ cells) infiltrate the exocrine pancreas compared with all other groups, other than sDMPanc. Reprasentative immunofluorescence confocal images of CgA (red), TH (grey), CD45 (green) and nuclei (blue) in (**E**) control dogs, (**F**) sDM, (**G**) sDMPanc, (**H**) sPanc and (**I**) iDM. P-values represented by * (P ≤ 0.5), ** (P ≤ 0.01), *** (P ≤ 0.001) and **** (P ≤ 0.0001).

To investigate the role of inflammation in the pathogenesis of canine DM and its potential association with loss of sympathetic innervation, we stained pancreata with TH, a rate-limiting enzyme of catecholamine biosynthesis and a marker of sympathenic innervation, and the pan-leukocyte cell surface marker CD45. Islet area was visualized by staining CgA. An early and sustained islet-selective loss of sympathetic innervation was shown previously in human patients with T1DM and models of autoimmune insulitis^11^. In all disease groups, TH surface area per islet was decreased compared to controls (Fig. 1A). The decrease in TH expression per islet was most extreme in the sDMPanc (90%, p < 0.0001), sDM (86%, p < 0.0001) and iDM (85%, p < 0.0001) but was also significant in the sPANC group (75%, p < 0.0001, Fig. 1A). While the mean size of islets, as determined by CgA positive area, was higher in control and sPanc dogs compared with sDM and sDMPanc dogs (433.2, 407.3, 117.9 and 215.7 µm^2^, respectively), changes were not statistically significant (Fig. 1B). The only group that had a significant reduction in islet size compared with controls (p < 0.01) and Panc (p < 0.01) was iDM (433.2, 407.3 and 19.98 µm^2^, respectively). After adjusting for loss of islet area (TH/CgA ratio), sDM, sDMPanc and sPANC groups were not different from the control group (Fig. 1C). TH/CgA ratio was significantly increased in the iDM group, suggesting that the decrease in TH expression per islet in iDM is likely secondary to marked endocrine cell loss and not due to loss of islet sympathetic innervation as in the other disease groups.

There were no intra-islet nor peri-islet CD45+ cells consistent with insulitis in controls, sDM, sDMPanc, sPanc or iDM. In the vicinity of the islets within the exocrine pancreas, there were rare CD45+ cells scattered in dogs with sDMPanc (0–40 cell/mm^2^) and in iDM (0–68 cells/mm^2^; Fig. 1D). This degree of immune cell infiltration falls well below the lowest category of inflammation consistent with pancreatitis^14^. The morphology of these cells was most consistent with lymphocytes. Representative images depicting CgA, TH and CD45 staining patterns in the various groups are included (Fig. 1E–I).

**Figure 2:**
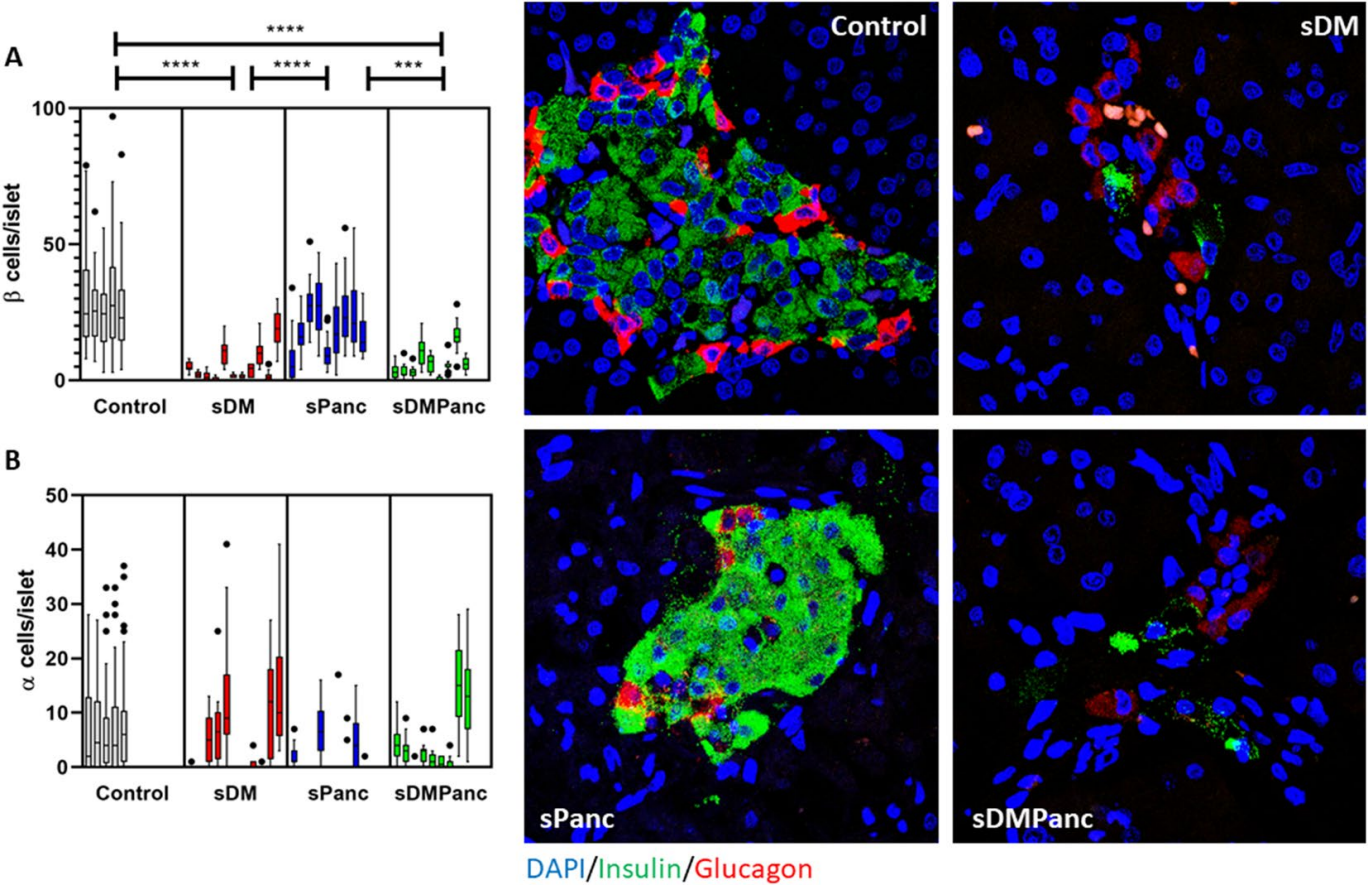
Selective β-cell loss in dogs with spontaneous diabetes mellitus (DM) and dogs with pancreatitis and concurent DM. *sDM* Spontaneous DM, *sDMPanc* spontaneous DM and concurrent pancreatitis, *sPanc* spontaneous pancreatitis. (**A**) Pancreatic β-cells are significantly reduced in numbers/islet in pancreata of dogs with spontaneous DM and dogs with pancreatitis and DM compared with normal control dogs and dogs with pancreatitis and no DM. (**B**) There is no significant difference in pancreatic α-cell numbers/islet between all groups. Reprasentative immunofluorescence confocal images of insulin (green), glucagon (red) and DNA (blue) representing β-cells, α-cells and nuclei, respectively, in pancreate from (**C**) normal control dogs compared to (**D**) dogs with DM**, (E**) DM and (**F**) pancreatitis and pancreatitis only. p-values represented by *** (P ≤ 0.001) and **** (P ≤ 0.0001).

We hypothesized that in dogs with pancreatitis, exocrine pancreatic inflammation induces indiscriminate collateral damage to islets. In order to test our hypothesis, we determined numbers of alpha cells and beta cells per islet via multicolor confocal microscopy in pancreas tissues from controls, sDM, sDMPanc, and sPanc. We hypothesized that if DM in the sDMPanc group is driven by indiscriminate collateral damage to islets, alpha cells and beta cells should be equally decreased and total islet area would be smaller compared to sDM without pancreatitis. We found that the number of beta cells per islet in sDM and sDMPanc dogs did not differ and both groups had decreased beta cells compared with control dogs (p < 0.0001) and compared with sPanc dogs (p < 0.001, Fig. 2A). The number of alpha cells per islet was not different in the sDM, sDMPanc and sPanc groups (Fig. 2B), suggesting that there is a selective beta cell loss in both the sDM and sDMPanc groups. Representative images of alpha and beta cell distribution within pancreatic islets in control dogs, sDM, sDMPanc and sPANC are depicted (Fig. 2C–F). Further supporting the hypothesis that there is similarly selective beta cell loss in sDM and sDMPanc, was the finding of a similar decrease in islet area (as represented by CgA area) in sDM and sDMPanc (Fig. 1A). Representative images of CgA area in control dogs, sDM, sDMPanc and sPANC are depicted (Fig. 1E–I). In human T1DM, a characteristic histological feature is the presence of islets which are entirely devoid of insulin across the pancreas. In contrast, this was not a common finding in dogs of this study. Iselts that are entirly devoid of insulin were observed in only 3 of the 11 dogs in the sDM group (31%, 35% and 85% of islets in each) while in three other dogs in this group, no islets were identified at all but clusters of 1–3 beta cells were scattered throughout the pancreas (Figure S1). Of the eight dogs in the sDMPanc group, three had islets completely devoid of insulin (6%, 7%, 16% of islets) and in a 4th dog of this group no islets were identified but clusters of 1–3 beta cells were scattered throughout the pancreas.

**Figure 3:**
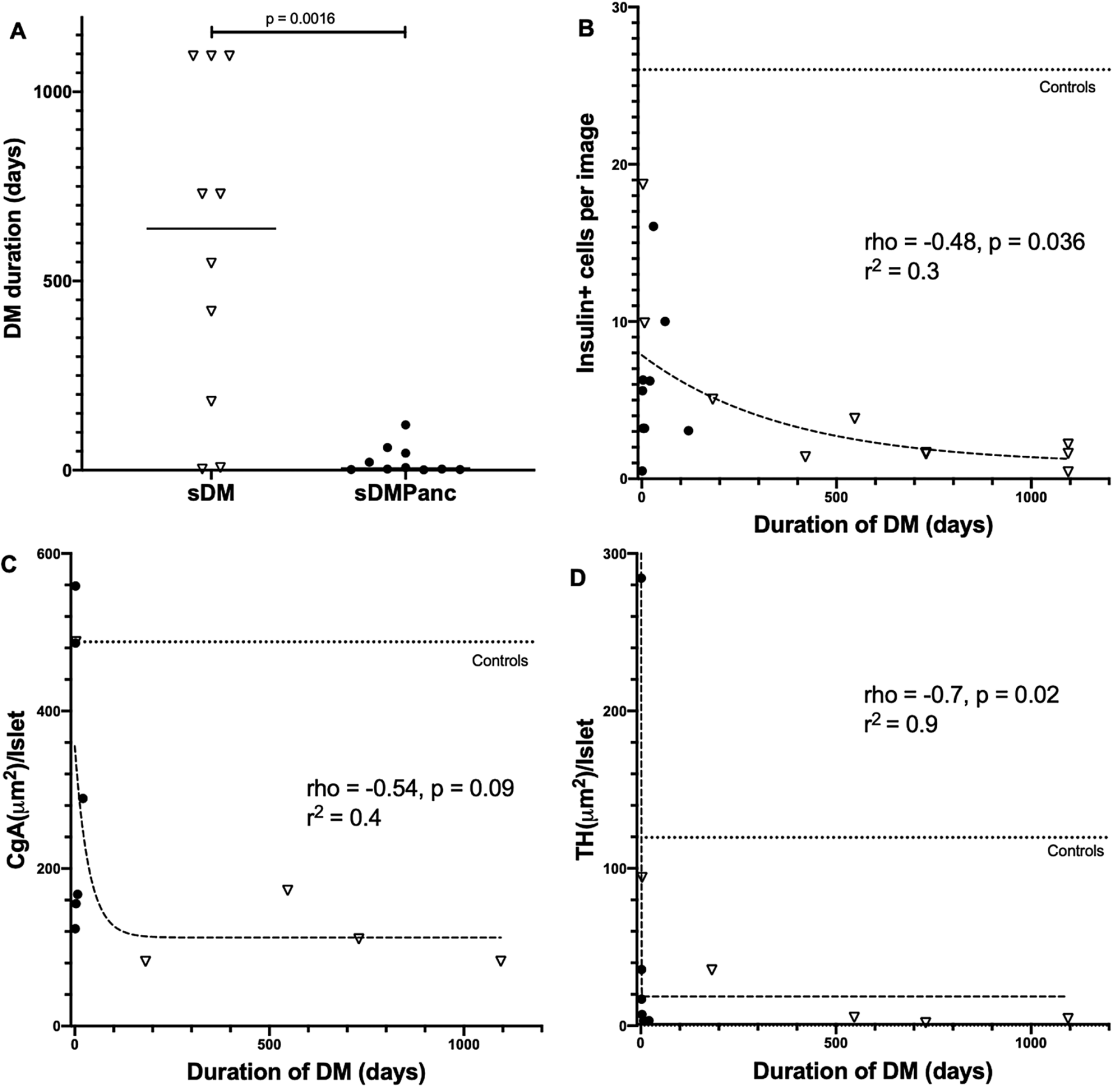
(**A**) Duration of diabetes mellitus (DM) in spontaneous DM without pancreatitis (sDM, open triangles) vs. sDM with concurrent pancreatitis (sDMPanc, closed circles). Horizontal solid line represents median. When all diabetic dogs were considered as one group (sDM, and SDMPanc), duration of DM is negatively corrleatd (spearman’s rho) with (**B**) beta cell numbers, (**C**) islet area (as expressed by Chromogranin A [CgA] area) and (**D**) sympathetic innervation as expressed by tyrosine hydroxylase (TH) area/islet. Dashed lines (with corresponding r^2^) represent best fit for one phase decay regression. Dotted lines represent median of healthy controls.

Median [range] DM duration was significantly shorter in sDMPanc (5 [1–120] days) compared to sDM (638 [3–1095] days, p = 0.0016, Fig. 3A). When sDM and sDMPanc were combined, beta cell numbers were negatively correlated with DM duration (rho = − 0.48, p = 0.036, Fig. 3B) and there was also a trend towards overall loss of islet area (represented by CgA expression, rho = − 0.54, p = 0.09, Fig. 3C) but there was no correlation between disease duration and alpha cell loss (p = 0.5, data not shown). With the progressive loss of beta cells and total islet area, there was also a strong negative correlation between TH area per islet and DM duration (rho = − 0.70, p = 0.02, Fig. 3D). These findings suggest that loss of sympathetic innervation is ongoing and does not precede beta cell loss as is the case when autoimmunity is the cause of DM^11–13^.

## Discussion

The etiology of canine DM is poorly understood, however, we report for the first time in this study on the sympathetic denervation of the islets of Langerhans as a key characteristic of this disease that helps narrow down possible etiologies. Overall, our histological assessment of pancreas pathology in dogs with DM does not reveal clear evidence of autoimmunity as the underlying cause of canine DM and suggests that the cause of selective beta cell loss should be further investigated. Canine DM is often proposed as a valuable, but overlooked, animal model of human T1D, and our work further highlights the need to deepen our understanding of the pathogenesis of this model^15^.

Sympathetic denervation of islets was reported in three animal models of autoimmune DM as well as in T1DM in people, but not in type 2 DM^11–13^, leading to the suggestion that this phenomenon is tightly linked with autoimmune insulitis. This phenomenon has been dubbed eSIN (early Sympathetic Islet Neuropathy)^16^ because in contrast to the classic Diabetic Autonomic Neuroparthy (DAN), it precedes the loss of beta cells. We showed that islet sympathetic denervation is a feature of canine DM, but also occurs in canine pancreatitis without DM, suggesting that at least in dogs, it is inflammation in general, not necessarily autoimmunity, that leads to islet neuropathy. Furthermore, in contrast to eSIN, we found evidence that in dogs, islet neuroparthy is ongoing, correlated to ongoing beta cells loss, and does not precede beta cell loss as in eSIN. These findings are important not only because they do not lend support to the autoimmune etiology hypothesis in canine DM but also because of their clinical implication. Islet neuropathy is associated with decreased glucagon responses in diabetics and inability to respond appropriately to insulin over-dosing which increases the risk and severity of life-threatening hypoglycemia^16^. Our finding that loss of sympathetic innervation is associated with beta cell numbers in canine DM suggests that the risk of hypoglycemia increases with disease duration in dogs. This hypothesis should be further tested, but if true, it would imply that glycemic targets should be modified in dogs based on disease duration, a strategy that is not currently employed in dogs.

Importantly, we demonstrated throughout our study that the pathological changes in islets of dogs with sDM do not differ from those of dogs with sDMPanc, increasing the likelihood that the etiology of DM in both groups is the same, and that pancreatitis is a complication in a subset of sDM dogs. We found a selective loss of beta cells in dogs with spontaneous DM, with and without pancreatitis, as was previously shown in dogs^8^. This was evident by sparing of alpha cells as well as by an overall similar reduction in islet size in sDM and SDMPanc. These data suggest that collateral damage from inflammation in the exocrine pancreas is not a major contributor to beta cell loss in these dogs, especially considering the absence of CD45 positive cells in or near islets.

Shields et al., looked for evidence of autoimmune insulitis in canine DM by staining for CD3+ lymphocytes^8^. However, insulitis in people is sometimes dominated by B lymphocytes (CD20+)^17^. By using CD45 as a pan-leukocyte marker and applying the current low-threshold standard for diagnosis of insulitis^10^, we were more likely than previously to detect insulitis. To minimize the risk of false positive staining associated with this greater sensitivity and to avoid misinterpreting “background noise” of inflammation or resident lymphocytes as insulitis, we used samples from dogs with pancreatitis and dogs with no pancreatitis as a control group to diabetic dogs. Strikingly, no pancreatic tissue and no single islet in our study met the low-threshold standard criteria for diagnosis of insulitis. In fact, we found no inflammatory cells at all within or adjacent to islets in control dogs, dogs with DM, and even in dogs with DM and pancreatitis. It has been hypothesized that the lack of evidence for autoimmunity in canine DM is related to the fact that the autoimmune process subsides when beta cells are no longer present in the islet^8^. This hypothesis might explain the apparent absence of insulitis in histopathological samples from diabetic dogs that died long after the diagnosis of the disease, but it has a few deficiencies: (1) we showed that after DM is clinically established in dogs, loss of beta cells continues and therefore, the cause of that ongoing loss should still be present. (2) Insulitis is also not observed in samples from dogs with early diagnosis of DM and when beta cells are still present in islets^8^. (3) Although early studies demonstrated the presence of antibodies to insulin or to beta cells in some diabetic dogs, more recent studies failed to replicate these findings and demonstrated no humoral response to either insulin or beta cells prior to insulin therapy^2,9,18^. It has been proposed previously that canine DM is pathogenically similar to Latent Autoimmune Diabetes of Adults (LADA), explaining its typical diagnosis in dogs at middle age or older and in the absence of insulitis^19^. However, even in LADA, at least 29% of human patients have evidence of insulitis, even when including older studies that have not used the current low-threshold standard^10,20^. As mentioned above, and even if ignoring the CD45 data subset, the pathology of islets in dogs with sDM and sDMPanc is similar. Combining these two groups, if canine DM was similar to LADA, we would have expected at least some of the cases in our study to demonstrate insulitis. Our data therefore do not support autoimmunity, acute or chronic, as a cause of canine DM.

In people, autoimmune DM is more commonly diagnosed in juveniles. Reports on DM in juvenile dogs (< 1 year old) are rare, and while they uniformly describe insulin-dependent dogs, they describe various histopathological abnormalities of the pancreas, with no clear etiology^2,8^. Only rarely, these reports support autoimmunity as a cause of juvenile DM^8^. Overall, less than 2.2% of all dogs diagnosed with DM are diagnosed before they are 1 year old, and still less than 3% of all dogs diagnosed with DM are diagnosed before they are 3 years old^1^. We therefore chose to exclude juvenile dogs from the DM groups in our study, because it is likely that their disease process does not represent the disease process in the majority of the population.

Gestational diabetes has been reported in dogs and is thought to be associated with progesterone-induced GH production in the mammary glands, leading to insulin resistance^21^. However, the prevalence of DM in intact females is not higher than the general canine population and being intact vs. spayed does not increase the risk of DM in a female dog^1^, suggesting that the underlying cause of DM in intact females is not different than in the rest of the population. Rather, it is likely that insulin resistance caused by progesterone-induced GH production exposes the underlying pancreatic lesion during diestrous. We therefore did not exclude intact females from our study.

One potential limitation of studies of patient-sourced tissues (from people or pets), especially retropesctive ones, is the inability to quantify the extent to which entire pancreata is affected by pancreatitis. Pancreatitis can be focal or multi-focal and therefore a single section of the organ might not represent the extent of organ tissue injury. As such, correlating histopathological lesions with function and clinical outcome may be discordant. Histopathological evidence of pancreatitis is found in most dogs that die from diseases unrelated to the pancreas and that do not present with clinical signs associated with pancreatitis. In one study, 63 of 70 (90%) dogs that died of a variety of causes had histopathological evidence of pancreatitis (acute and chronic) although only 17 had biochemical evidence of pancreatitis (increased serum pancreatic lipase) and only 11 had clinical signs consistent with pancreatitis^14^. In an earlier study, 73 pancreata were collected consecutively from dogs that died from a variety of causes and 47 of them (64.4%) had histopathological evidence of pancreatitis^22^. Therefore, the clinical significance of the histopathological diagnosis of pancreatitis remains unknown. In both of these studies, diagnosis of pancreatitis was based on systematic serial sectioning of the entire pancreas (every 2 cm) and standard scoring of each section. Dogs were then diagnosed with pancreatitis if lesions were observed in any of the sections, regardless of distribution throughout the entire organ. We used tissues from one of these studies^14^ and selected only cases that had no evidence of pancreatitis on any of the section, therefore confirming the appropriatenss of categorizing these cases as controls for our study. This systematic approach, however, is not routinely implemented in clinical cases (human and veterinary). Therefore, in most cases available to us, only a single location of the pancreas was sectioned based on ad-hoc judement of the attending pathologist, and the presence or absence of pancreatitis was based on these single tissue section.

In conclusion, we found no evidence to support the hypothesis that selective beta cell loss in canine DM is caused by autoimmunity or by collateral damage from pancreatitis. The decrease in sympathetic innervation to islets in non-diabetic dogs suffering from pancreatitis suggests that pancreatitis might be a precursor to islet pathology by yet unknown mechanisms that should be further explored.

## Material and methods

### Animals

All animal studies perfomed at UC Davis in this study were approved by the Institutional Animal Care and Use Committee (IACUC protocol #21100) and followed IACUC guidelines. All pancreatic tissue samples in this study were collected from archived tissues at the UC Davis William R. Pritchard Veterinary Medical Teaching Hospital (VMTH) Pathology laboratory, except for samples from dogs with induced DM (see below). Study cases were identified by searching the electronic medical records at UCD VMTH for diagnoses of DM and pancreatitis in dogs from 1988 to 2016. The selected cases were recorded in an Excel spreadsheet and were divided into five groups: Group 1 (Control, N = 7) was of non-diabetic dogs that in a previous study were confirmed to have no clinical and histopathologic evidence of pancreatic disease^14^. Specifically, in each dog, for each limb of the pancreas, serial transverse sections were made every 2 cm throughout the pancreas, and each individual section was examined and scored for acute and chronic inflammation on H&E using a semiquantitative histopathologic grading scheme by a board certified veterinary pathologist as previously described^14^. In brief, nine histopathologic parameters were evaluated including suppurative inflammation, acinar cell necrosis, hemorrhage, interstitial edema, peripancreatic fat necrosis, mononuclear inflammation, fibrosis, atrophy, and cellular degeneration. Each transverse section was individually scored for all nine parameters on a scale from 0 to 3. A score of 0 indicated none of the sectional surface area was affected by that particular histopathologic change. Scores of 1, 2 and 3 had < 10%, 10–40% and > 40% of the examined surface area affected by the specific lesion parameter, respectively. Additional histopathologic changes not captured by this scoring system were also recorded for each pancreas (e.g. nodular hyperplasia and neoplasia). Dogs were included in the control group only if their cumulative score was 0. Group 2 represented dogs that were diagnosed with spontaneous DM without pancreatitis (sDM, N = 11); group 3 dogs were diagnosed with spontaneous DM and spontaneous pancreatitis (sDMPanc group, N = 10); group 4 dogs were diagnosed with spontaneous pancreatitis but not with DM (sPanc, N = 10). Inclusion into groups 3 and 4 required a standard histological diagnosis (including the nine histologic parameters specified above) by a board certified veterinary pathologist. Group 5 dogs were purpose-bred Beagle dogs with alloxan-streptozotocin induced DM (iDM, N = 7). For each case, the following clinical data was extracted from the medical record: age, sex, breed, body weight, histopathologic diagnosis and duration of DM.

Cases were excluded if they did not have a necropsy report, were clinically diagnosed with pancreatitis but the diagnosis was not confirmed histopathologically, had an autolyzed postmortem body condition, or did not have any archived pancreatic tissue. Dogs under the age of 4 years were also excluded from the spontaneous DM (sDM) and sDM + spontaneous pancreatitis (sDMPanc) groups.

Induction of DM was achieved as previously described ^23^ at Sinclair Research LLC, as part of a different study which was approved by institutional ethical committee. Dogs were induced 8 months before tissue. All procedures performed at UC Davis on these dogs were approved by the Institutional Animal Care and Use Committee (IACUC protocol #21100). Futher details on the husbendary of iDM dogs are available in “Supplementary Methods”.

### Tissue processing, image acquisition and image analysis

All pancreatic tissues were fixed in 10% neutral buffered formalin and embedded in paraffin blocks. Four µm sections were cut from each pancreatic tissue block. Tissue sections were prepared routinely for multiplex immunofluorescence staining. Primary antibodies targeting insulin, glucagon, chromogranin A (CgA), tyrosine hydroxylase (TH) and CD45 were used (Tables S1 and S2). All immunohistochemistry and immunofluorescence studies had several layers of negative and positive controls to ensure appropriate interpretation of our findings. A Leica TCS SP8 STED 3X confocal laser scanning microscope was used to take 63× Z-stack images of islets and a 20× stitched image of the canine pancreatic section. For each image, ImageJ (NIH, Bethesda, MA) was used to measure the following when applicable: Total Image Surface Area, Number of Islets, CgA Area of Islet(s), TH Area of Islet(s), total CD45+ cells, and %TH relative to %CgA. Futher details on tissue processing, image acquisition and image analysis are available in “Supplamentary Methods”.

### Statistical analysis

Statistical analysis was performed using a commercially available computer software (GraphPad Prism, version 8.0; GraphPad Software Inc, La Jolla, CA). Visual inspection of the data and the Shapiro–Wilk test were used to assess normal distribution. Demographic data are presented as mean ± SD and median (range). For distribution of beta cells, alpha cells, TH area, CgA area, and CD45+ cells, nested tables were created and a non-parametric 1-way ANOVA was applied to compare between disease groups after removal of outlier values (ROUT method, Q = 0.5%). Adjusted p values are presented for post-hoc multiple comparisons. Duration of disease was not normally distributed and was compared between sDM and SDMPanc using the Mann–Whitney test. For the association between duration of disease and beta cells, TH area, and islet area, a non-linear correlation (Spearman’s rho) was calculated, and a regression model of one-phase decay was fit. Significance was set at p ≤ 0.05.

## Supplementary information

Supplementary Information 1.

Supplementary Information 2.

Supplementary Information 3.

## Data Availability

The datasets generated during and/or analysed during the current study are available from the corresponding author on reasonable request.
