# Supplementary material for: Loss of sympathetic innervation to islets of Langerhans in canine diabetes and pancreatitis is not associated with insulitis
Source: Sci Rep. 2020 Nov 5;10:19187. doi: 10.1038/s41598-020-76091-5 (PMC7645777; doi:10.1038/s41598-020-76091-5)
Supplement: Supplementary file 1 — Supplementary Information 1. [file 41598_2020_76091_MOESM1_ESM.docx]

**Figure S1. Variation in β-cell number and morphology in islets of dogs with sDM.** Spontaneous DM = sDM. Confocal immunofluorescence (panel A-F) highlighting the spectrum of β-cell number and morphology in islets from with sDM. β-cells loss ranges from islets that are completely devoided of β-cells (A), through islets that have varying degrees of residual (though still markedly decreased) β-cells (B-F). Many of the residual β-cells have an abnormal and punctate staining pattern suggesting β-cell injury.
