# Supplementary figures and images for: Loss of sympathetic innervation to islets of Langerhans in canine diabetes and pancreatitis is not associated with insulitis
Source: Sci Rep. 2020 Nov 5;10:19187. doi: 10.1038/s41598-020-76091-5 (PMC7645777; doi:10.1038/s41598-020-76091-5)

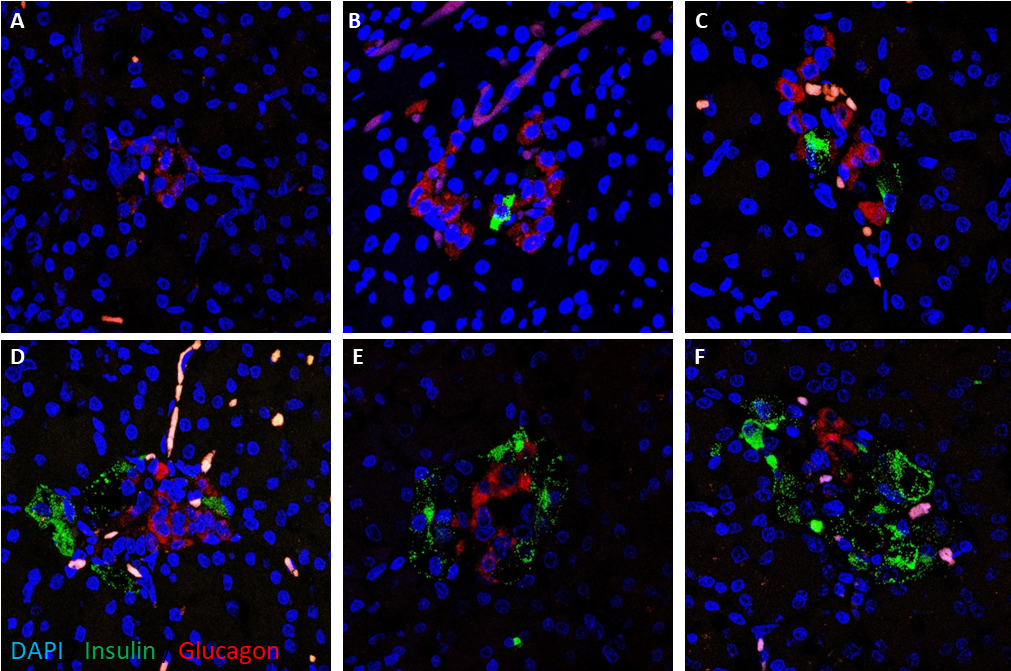

Supplement: Supplementary file 2 — Supplementary Information 2. [file 41598_2020_76091_MOESM2_ESM.tif]
