# Supplementary material for: Loss of sympathetic innervation to islets of Langerhans in canine diabetes and pancreatitis is not associated with insulitis
Source: Sci Rep. 2020 Nov 5;10:19187. doi: 10.1038/s41598-020-76091-5 (PMC7645777; doi:10.1038/s41598-020-76091-5)
Supplement: Supplementary file 3 — Supplementary Information 3. [file 41598_2020_76091_MOESM3_ESM.pdf]

Loss of sympathetic innervation to islets of Langerhans in canine diabetes and pancreatitis is not associated with insulitis

Supplemental Materials

Chen Gilor,[a,b*] Jully Pires,[a] Rachel Greathouse,[c] Rebeca Horn,[c] Mark O. Huising,[d] Stanley L. Marks,[a] Brian Murphy,[c] Amir Kol.[c*]

Animals

Study cases were identified by searching the electronic medical records at UCD VMTH for diagnoses of DM and pancreatitis in dogs from 1988-2016. To confirm that our control group did not have any pancreatic lesion, in each dog, for each limb of the pancreas, serial transverse sections were made every 2 cm throughout the pancreas, and each individual section was examined and scored for inflammation on H&E [14]. For each dog in all of the study groups, the following clinical data was extracted from the medical record: age, sex, breed (Table S1), body weight, histopathologic diagnosis and duration of DM. Cases were excluded if they did not have a necropsy report, were clinically diagnosed with pancreatitis but the diagnosis was not confirmed histopathologically, had an autolyzed postmortem body condition, or did not have any archived pancreatic tissue. Dogs under the age of 4 years were also excluded from the spontaneous DM (sDM) and sDM + spontaneous pancreatitis (sDMPanc) groups.

Induction of DM was achieved as previously described [15] at Sinclair Research LLC, as part of a different study which was approved by Institutional Animal Care and Use Committee. Dogs were induced 8 months before tissue collection and 3 months before dogs were transferred to UC Davis (UC Davis IACUC protocol #21100). These dogs were housed individually with free access to drinking water and supervised access to outdoor play pens. During the 8 months

after DM induction, dogs were treated with various insulin formulations with no other intervention (other than routine healthcare preventatives including anthelmintic medications and standard anti-viral vaccines; no antibiotics were administered). Body weight and general health assessment were performed weekly and throughout the 6 month period prior to sampling. All animals maintained their body weight and ideal body condition score with no clinical evidence of gastrointestinal disease or pancreatic disease other than DM. Dogs were monitored daily and fed a standard canine laboratory chow of 300g/day throughout the course of the study to meet their energy requirement. Three weeks prior to tissue harvesting, dogs were treated with fenofibrate (Tricor®) at a dose of 10mg/kg orally once daily for 3 weeks, as part of a different study. They were then euthanized and pancreata were collected within 30 minutes.

Tissue processing

Formalin fixed and paraffin embedded tissue sections were routinely deparaffinized in xylene and serial ethanol dilutions, following heat-induced antigen retrieval (antigen retrieval buffer; Dako). Samples were further blocked with normal donkey serum and FcR Blocking Reagent (Miltenyi) and incubated overnight with the appropriate primary antibodies (Table S2). Two multiplex panels were used: 1) insulin-glucagon and 2) chromogranin A (CgA)-tyrosine hydroxylase (TH)-CD45. Twenty-four hours later, slides were extensively washed and treated with the appropriate set of secondary antibodies (Table S32). Finally, nuclei were labeled with DAPI (ThermoFischer Scientific). All immunohistochemistry and immunofluorescence studies had several layers of negative and positive controls to ensure appropriate interpretation of our findings. Specifically, we used a 'No antibody' control to determine the level of tissue auto-fluorescence and 'No primary antibody control' to determine the level of non-specific secondary antibody binding. For CD45 staining, canine gut and lymph node tissues were used as positive

controls. All pancreatic tissue sections had TH staining within the exocrine pancreas serving as positive control.

Image acquisition

Each group (Controls, sDM, sDMPanc, sPanc and iDM) had optimized image settings applied across the entire group. To mitigate any bias in field selection for TH and CD45 analysis, prior to looking at the 20x stitched image, the Alexa Fluor-488 and Cy3 channels were turned off. DAPI and Alexa Fluor-647 (CgA) remained visible as the 20x stitched image was scanned and acquired. Once the 20x field of view was set, the tissue was divided into 16 quadrants. With only DAPI and CgA visible, a representative islet was selected for high power Z-stach imaging in each quadrant: In a quadrant with several small islets, a small islet would be selected while in a quadrant with mostly large islets, a large islet would be selected. For the purpose of image analysis, we defined islets as a cluster of 3 or more CgA+ cells. If there were no islets available to image, areas with less than 3 CgA+ cells in a cluster were imaged and labeled as 'endocrine cells' instead of 'islets'. Given the small size of some tissue samples, some of the quadrants displayed only empty spaces. To be able to exclude empty spaces from analysis, only 12 of the 16 quadrants per tissue were selected for imaging to set consistency across all groups of tissues. Only after 12 imaging areas were selected, the TH channel was made visible to avoid cutting off TH signal associated with the pre-selected islets. Subsequently, selected islets in quadrants were imaged at 63x and z-stacked according to tissue thickness.

Image analysis

Quantitative image analysis was performed on 63x images using ImageJ (NIH, Bethesda, MA). There were 4 total channels involved in the analysis: Nuclei (DAPI), TH (Cy3), CD45

(Alexa Flour 488), and CgA (Alexa Flour 647). Macro scripts were applied across all images to convert them into TIFF files, convert the z-stacks into z-projects, automate background noise reduction and remove autofluorescence. Thresholding the TH and CgA signal was performed in the same manner. After a composite image was created using the optimized channel settings, CD45+ cells were identified and counted.

Table S1. Primary antibodies

**Target antigen**	**Target Species**	**Host species**	**Monoclonal / polyclonal**	**Dilution used**	**Make**	**Catalogue number**
Chromogranin A	Human	Goat	Polyclonal	1:100	Ray Biotech	119-14358
Insulin	Human	Guinea pig	Polyclonal	1:500	Dako	A0564
Glucagon	Human	Rabbit	polyclonal	1:500	Cell Signaling Technology	2760
Tyrosine hydroxylase	Rat	Rabbit	Polyclonal	1:100	Pel Freez Biologicals	P40101-150
CD45	Canine	Mouse	Monoclonal (CA12.10C12)	1:5	Bio-Rad	MCA2035S

Table S2. Secondary antibodies

**Host species**	**Target species**	**Fluorophore**	**Dilution used**	**Make**
Donkey	Guinea pig	Alexa Fluor 488	1:400	Life Science
Donkey	Rabbit	Alexa Fluor 647	1:400	Life Science
Donkey	Mouse	Cy3	1:600	Jackson ImmunoResearch
Donkey	Mouse	Alexa Fluor 488	1:400	Life Sciemce
Donkey	Goat	Cy3	1:500	Jackson ImmunoResearch

Table S3. Dog breed ditribution. DM = Diabetes mellitus; spontaneous DM = sDM; spontaneous DM and concurrent pancreatitis = sDMPanc; spontaneous pancreatitis = sPanc; toxin-induced DM = iDM

Group	Number of mixed breed dogs	Other breeds
Controls	1	1 Pit Bull Terrier, 1 Brittany Spaniel, 1 Great Pyrenees, 1 Chinese Crested
sDM	2	2 Labrador Retrievers, 1 Cocker Spaniel, 1 Springer Spaniel, 1 Golden Retriever, 1 Alaskan Malamute, 1 American Eskimo, 1 Miniature Poodle, 1 Miniature Schnauzer
sPanc	7	1 Golden Retriever, 1 Boxer, 1 Beagle
sDMPanc	4	2 Miniature Schnauzer, 1 Welsh Corgi, 1 Rottweiler, 1 Australian Terrier, 1 Miniature Poodle;
iDM	0	7 Beagles
